# Supplementary material for: Antiproliferative Effect of Colonic Fermented Phenolic Compounds from Jaboticaba (Myrciaria trunciflora) Fruit Peel in a 3D Cell Model of Colorectal Cancer
Source: Molecules. 2021 Jul 24;26(15):4469. doi: 10.3390/molecules26154469 (PMC8347777; doi:10.3390/molecules26154469)

T**able S1**: The loadings of the first three principal components.

**VARIABLE NUMBER**	**PHENOLIC COMPOUND**	**PC1 (51.5%)**	**PC2 (25.1%)**	**PC3 (14.7%)**
**1**	HHDP-galloylglucose; Monogalloyl glucose	**0,7690**	**−0.46936**	0.13524
**2**	Digalloylglucose; Gallic acid	**0.86956**	−0.27585	0.09153
**3**	HHDP-galloylglucose and bis-HHDP-glucose	**0,9047**	−0.11130	0.06778
**4**	Digalloylglucose isomer	**0.88635**	−0.06148	0.06996
**5**	Protocatechuic acid	**0.59064**	**−0.68325**	0.10748
**6**	Bis-HHDP-glucose isomer	**0.61709**	**−0.54739**	**0.31575**
**7**	Castalin; HHDP-digalloylglucose	**0.82617**	**−0.39079**	0.10511
**8**	Castalin isomer and (Epi)catechin	**0.84344**	**−0.33137**	−0.11529
**9**	HHDP-galloylglucose	**0.85304**	−0.23464	0.09121
**10**	Digalloylglucose isomer	**0.52257**	**−0.63127**	−0.26288
**11**	Trigalloylglucose	0.19198	0.24590	**0.51143**
**12**	HHDP-digalloylglucose isomer +Dihydroxyphenyl-c-valerolactone	0.13844	**−0.62458**	**0.38224**
**13**	Trigalloylglucose isomer	**0.83354**	0.26437	−0.02877
**14**	Castalin/Vescalagin	**0.76761**	**−0.41498**	0.16745
**15**	Trigalloylglucose isomer	**0.86305**	0.17555	0.00100
**16**	Tetragalloylglucose	**0.8949**	0.05313	−0.06685
**17**	Tetragalloylglucose isomer	**0.60034**	**0.57494**	−0.15656
**18**	Pentagalloyl glucose	**0.58634**	**0.58866**	−0.13905
**19**	Galloyl-castalagin	**−0.87842**	0.18995	0.18671
**20**	Urolithin B sulfate	−**0.88001**	0.04832	0.27510
**21**	Urolithin M5	**−0.77249**	0.11761	**0.47313**
**22**	Ellagic acid hexoside	**−0.34766**	**0.73047**	**−0.30215**
**23**	Urolithin D	**−0.78209**	0.18306	**0.41769**
**24**	Ellagic acid pentoside	**−0.69168**	**0.46002**	**0.30514**
**25**	Urolithin D isomer	−**0.88660**	0.17430	0.14346
**26**	Urolithin M6	**−0.89495**	0.15064	0.26448
**27**	Ellagic acid pentoside+ Ellagic acid	**0.85813**	−0.22747	−0.22642
**28**	Myricetin-rhamnoside	**0.79219**	**0.34589**	−0.05550
**29**	Quercetin-hexoside A	**0.86336**	0.17255	−0.01422
**30**	Urolithin C	**−0.87136**	0.16382	−0.06242
**31**	Quercetin-hexoside B	**0.88519**	0.05063	0.03698
**32**	Quercetin-pentoside A	**0.58578**	**0.58747**	−0.13876
**33**	Urolithin M7	**−0.87445**	0.21726	0.21334
**34**	Quercetin-pentoside B	**0.50583**	**0.62490**	0.10516
**35**	Quercetin-rhaminoside	**0.85452**	−0.23057	0.11788
**36**	Isourolithin A	**−0.73443**	**0.43797**	−0.24396
**37**	Urolithin A	**−0.87266**	0.17553	0.25969
**38**	Delphinidin-3-glucoside	**0.59884**	**0.58836**	−0.14303
**39**	Cyanidin-3-glucoside	**0.72090**	**0.53149**	−0.11479
**40**	Peonidin-3-glucoside	**0.63356**	**0.54796**	−0.12428
**41**	Cell growth inhibition (%)	−0.11370	−0.16921	**−0.28772**

Bold font indicates $p < 0.05$.

Figure S1: Effect of FJPP (10.000 µg mL^{-1}) on viability of Caco-2 cells. Cell viability was evaluated after exposure of Caco-2 cells to FJPP at 10.000 µg mL^{-1} for 72 h. Results are means of at least 6 independent experiments performed in triplicate ± SEM.

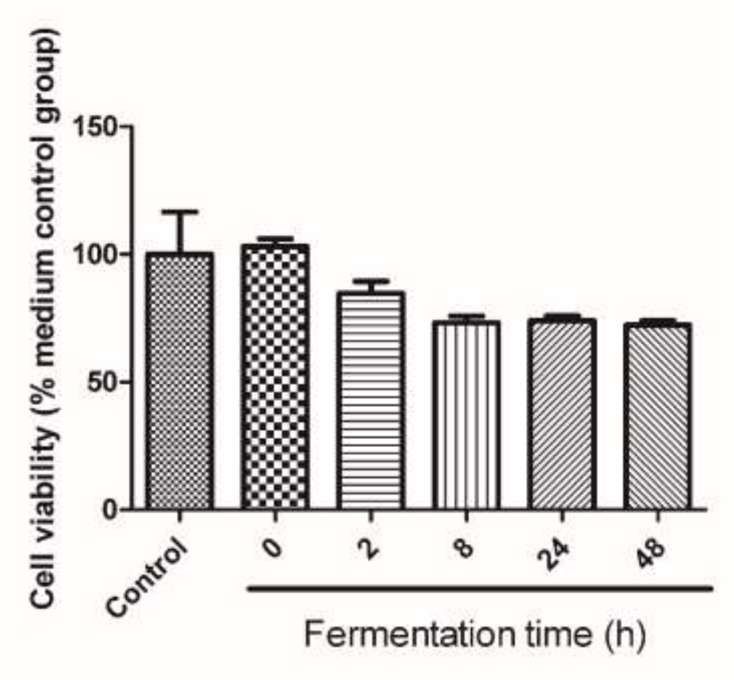

Supplement: Supplementary file 1 [file molecules-26-04469-s001.zip › molecules-1279564-supplementary.pdf]
